# Supplementary material for: Modeling glioblastoma heterogeneity as a dynamic network of cell states
Source: Mol Syst Biol. 2021 Sep 16;17(9):e10105. doi: 10.15252/msb.202010105 (PMC8444284; doi:10.15252/msb.202010105)
Supplement: Supplementary file 6 — Source Data for Figure 5 [file MSB-17-e10105-s004.zip › Figure5A_sourcedata/GSEA_3071/nosigdb_stateI.GseaPreranked.1622795145664/GAROFANO_IAVARONE_LIST_OF_NEURAL_PROGENITOR_MARKER_GENES.html]

Details for gene set GAROFANO\_IAVARONE\_LIST\_OF\_NEURAL\_PROGENITOR\_MARKER\_GENES[GSEA]

|  || Dataset | state13071 |
| Phenotype | NoPhenotypeAvailable |
| Upregulated in class | na\_neg |
| GeneSet | GAROFANO\_IAVARONE\_LIST\_OF\_NEURAL\_PROGENITOR\_MARKER\_GENES |
| Enrichment Score (ES) | -0.58666563 |
| Normalized Enrichment Score (NES) | -2.920537 |
| Nominal p-value | 0.0 |
| FDR q-value | 0.0 |
| FWER p-Value | 0.0 |
Table: GSEA Results Summary

  

Fig 1: Enrichment plot: GAROFANO\_IAVARONE\_LIST\_OF\_NEURAL\_PROGENITOR\_MARKER\_GENES      
 Profile of the Running ES Score & Positions of GeneSet Members on the Rank Ordered List

  

| PROBE | GENE SYMBOL | GENE\_TITLE | RANK IN GENE LIST | RANK METRIC SCORE | RUNNING ES | CORE ENRICHMENT || 1 | NOTCH2 |  |  | 446 | 0.551 | -0.1201 | No |
| 2 | GLI1 |  |  | 452 | 0.547 | -0.1119 | No |
| 3 | CLIC4 |  |  | 800 | 0.382 | -0.2061 | No |
| 4 | FOXA2 |  |  | 827 | 0.373 | -0.2071 | No |
| 5 | VIM |  |  | 1105 | 0.305 | -0.2824 | No |
| 6 | IRX3 |  |  | 1177 | 0.293 | -0.2978 | No |
| 7 | GLI2 |  |  | 1401 | 0.252 | -0.3583 | No |
| 8 | SAE1 |  |  | 1638 | -0.273 | -0.4222 | No |
| 9 | MIS18BP1 |  |  | 1764 | -0.289 | -0.4535 | No |
| 10 | NOTCH1 |  |  | 1878 | -0.301 | -0.4810 | No |
| 11 | MSI1 |  |  | 1888 | -0.303 | -0.4783 | No |
| 12 | PTBP1 |  |  | 1934 | -0.311 | -0.4859 | No |
| 13 | KPNA2 |  |  | 1944 | -0.312 | -0.4830 | No |
| 14 | SUZ12 |  |  | 2011 | -0.318 | -0.4966 | No |
| 15 | AHCY |  |  | 2089 | -0.333 | -0.5131 | No |
| 16 | NDE1 |  |  | 2111 | -0.337 | -0.5133 | No |
| 17 | RANGAP1 |  |  | 2207 | -0.354 | -0.5347 | No |
| 18 | NUCKS1 |  |  | 2278 | -0.368 | -0.5485 | No |
| 19 | TUBB4B |  |  | 2291 | -0.370 | -0.5455 | No |
| 20 | CKS1B |  |  | 2332 | -0.380 | -0.5504 | No |
| 21 | CKAP2 |  |  | 2357 | -0.385 | -0.5506 | No |
| 22 | UHRF1 |  |  | 2404 | -0.393 | -0.5570 | No |
| 23 | H2AFV |  |  | 2431 | -0.398 | -0.5576 | No |
| 24 | CKS2 |  |  | 2466 | -0.404 | -0.5603 | No |
| 25 | SPAG5 |  |  | 2519 | -0.418 | -0.5681 | No |
| 26 | RACGAP1 |  |  | 2522 | -0.419 | -0.5612 | No |
| 27 | PAICS |  |  | 2545 | -0.426 | -0.5601 | No |
| 28 | RPL22L1 |  |  | 2576 | -0.434 | -0.5612 | No |
| 29 | ANP32B |  |  | 2593 | -0.438 | -0.5581 | No |
| 30 | PAX6 |  |  | 2688 | -0.469 | -0.5772 | No |
| 31 | ANLN |  |  | 2702 | -0.472 | -0.5726 | No |
| 32 | PRC1 |  |  | 2705 | -0.472 | -0.5648 | No |
| 33 | NUF2 |  |  | 2781 | -0.497 | -0.5779 | Yes |
| 34 | TUBA1B |  |  | 2785 | -0.498 | -0.5699 | Yes |
| 35 | SLBP |  |  | 2792 | -0.499 | -0.5629 | Yes |
| 36 | LIG1 |  |  | 2848 | -0.520 | -0.5697 | Yes |
| 37 | CKAP2L |  |  | 2857 | -0.523 | -0.5627 | Yes |
| 38 | RAD21 |  |  | 2866 | -0.527 | -0.5558 | Yes |
| 39 | ANP32E |  |  | 2877 | -0.530 | -0.5493 | Yes |
| 40 | ARX |  |  | 2898 | -0.542 | -0.5455 | Yes |
| 41 | ARHGAP11A |  |  | 2921 | -0.556 | -0.5421 | Yes |
| 42 | SFRP1 |  |  | 2926 | -0.559 | -0.5334 | Yes |
| 43 | TPX2 |  |  | 3006 | -0.606 | -0.5457 | Yes |
| 44 | TCF19 |  |  | 3019 | -0.613 | -0.5383 | Yes |
| 45 | DLG1 |  |  | 3043 | -0.628 | -0.5339 | Yes |
| 46 | TMPO |  |  | 3088 | -0.666 | -0.5349 | Yes |
| 47 | NASP |  |  | 3106 | -0.682 | -0.5278 | Yes |
| 48 | DBI |  |  | 3165 | -0.746 | -0.5315 | Yes |
| 49 | DNAJC9 |  |  | 3167 | -0.747 | -0.5186 | Yes |
| 50 | OXCT1 |  |  | 3176 | -0.756 | -0.5075 | Yes |
| 51 | SALL1 |  |  | 3190 | -0.768 | -0.4977 | Yes |
| 52 | TCF12 |  |  | 3191 | -0.769 | -0.4841 | Yes |
| 53 | FBXO5 |  |  | 3212 | -0.797 | -0.4758 | Yes |
| 54 | RRM1 |  |  | 3215 | -0.801 | -0.4622 | Yes |
| 55 | UBE2C |  |  | 3225 | -0.812 | -0.4505 | Yes |
| 56 | HMGN2 |  |  | 3242 | -0.833 | -0.4404 | Yes |
| 57 | MYO10 |  |  | 3255 | -0.857 | -0.4288 | Yes |
| 58 | SOX9 |  |  | 3271 | -0.883 | -0.4175 | Yes |
| 59 | TP53I11 |  |  | 3315 | -0.952 | -0.4132 | Yes |
| 60 | EZH2 |  |  | 3342 | -1.015 | -0.4028 | Yes |
| 61 | DEK |  |  | 3344 | -1.025 | -0.3850 | Yes |
| 62 | PROM1 |  |  | 3347 | -1.035 | -0.3672 | Yes |
| 63 | PBK |  |  | 3354 | -1.046 | -0.3505 | Yes |
| 64 | ESCO2 |  |  | 3367 | -1.088 | -0.3347 | Yes |
| 65 | DLL3 |  |  | 3368 | -1.090 | -0.3154 | Yes |
| 66 | DHFR |  |  | 3387 | -1.159 | -0.3002 | Yes |
| 67 | HMGB2 |  |  | 3390 | -1.184 | -0.2798 | Yes |
| 68 | SOX2 |  |  | 3394 | -1.198 | -0.2595 | Yes |
| 69 | RRM2 |  |  | 3420 | -1.297 | -0.2438 | Yes |
| 70 | CLU |  |  | 3424 | -1.329 | -0.2212 | Yes |
| 71 | HES6 |  |  | 3431 | -1.366 | -0.1988 | Yes |
| 72 | NBL1 |  |  | 3432 | -1.368 | -0.1746 | Yes |
| 73 | INSM1 |  |  | 3483 | -1.929 | -0.1550 | Yes |
| 74 | ASCL1 |  |  | 3484 | -1.937 | -0.1208 | Yes |
| 75 | POU3F2 |  |  | 3487 | -2.092 | -0.0844 | Yes |
| 76 | FABP7 |  |  | 3496 | -2.308 | -0.0459 | Yes |
| 77 | GPD1 |  |  | 3506 | -2.825 | 0.0015 | Yes |
Table: GSEA details [plain text format]

  

Fig 2: GAROFANO\_IAVARONE\_LIST\_OF\_NEURAL\_PROGENITOR\_MARKER\_GENES: Random ES distribution      
 Gene set null distribution of ES for **GAROFANO\_IAVARONE\_LIST\_OF\_NEURAL\_PROGENITOR\_MARKER\_GENES**
